# Prenatal maternal depression is associated with offspring inflammation at 25 years: a prospective longitudinal cohort study

**DOI:** 10.1038/tp.2015.155

**Published:** 2016-11-01

**Authors:** D T Plant, S Pawlby, D Sharp, P A Zunszain, C M Pariante

**Affiliations:** 1 Department of Psychological Medicine, Institute of Psychiatry, Psychology and Neuroscience, King's College London, London, UK; 2 School of Social and Community Medicine, University of Bristol, Bristol, UK

## Abstract

Animal studies and a handful of prospective human studies have demonstrated that young offspring exposed to maternal prenatal stress show abnormalities in immune parameters and hypothalamic–pituitary–adrenal (HPA) axis function. No study has examined the effect of maternal prenatal depression on offspring inflammation and HPA axis activity in adulthood, nor the putative role of child maltreatment in inducing these abnormalities. High-sensitivity C-reactive protein (hs-CRP) and awakening cortisol were measured at age 25 in 103 young-adult offspring of the South London Child Development Study (SLCDS), a prospective longitudinal birth cohort of mother–offspring dyads recruited in pregnancy in 1986. Maternal prenatal depression was assessed in pregnancy at 20 and 36 weeks; offspring child maltreatment (birth 17 years) was assessed at offspring ages 11, 16 and 25; and offspring adulthood depression (18–25 years) was assessed at age 25. Exposure to maternal prenatal depression predicted significantly elevated offspring hs-CRP at age 25 (odds ratio=11.8, 95% confidence interval (CI) (1.1, 127.0), *P*=0.041), independently of child maltreatment and adulthood depression, known risk factors for adulthood inflammation. In contrast, maternal prenatal depression did not predict changes in offspring adulthood cortisol; however, offspring exposure to child maltreatment did, and was associated with elevated awakening cortisol levels (*B*=161.9, 95% CI (45.4, 278.4), *P*=0.007). Fetal exposure to maternal depression during pregnancy has effects on immune function that persist for up to a quarter of a century after birth. Findings are consistent with the developmental origins of health and disease (DOHaD) hypothesis for the biological embedding of gestational psychosocial adversity into vulnerability for future physical and mental illness.

## Introduction

There is mounting evidence that many psychiatric and physical health conditions originate in adverse early-life experiences, but the exact timing for the embedding of such adverse experiences into the individual's pathophysiology remains unknown, especially in regards to the relative contributions of prenatal and childhood environments. Much contemporary research has investigated the relationship between very early-life adversity, namely exposure to maternal prenatal stress, and neurodevelopmental and health outcomes in later life, including emotional, behavioral and cognitive psychopathology, stress physiology, brain plasticity, immune function and chronic metabolic diseases.^1, 2, 3, 4, 5, 6, 7, 8^ This body of research draws upon the theoretical premise of fetal programming, which posits that exposure to an adverse intrauterine environment, especially elevated levels of maternal glucocorticoids, can generate persistent changes in fetal biological systems, which subsequently confer risk for developmental disorders later in life.^9, 10, 11, 12^

A consistent clinical finding in the literature is that maternal prenatal depression predicts offspring depression across childhood and early adulthood.^13, 14, 15^ This has stimulated research into the putative biological mechanisms underlying this association, especially with reference to two prominent biological models of the pathogenesis of depression, specifically the inflammatory and the hypothalamic–pituitary–adrenal (HPA) axis systems.^16, 17, 18, 19, 20, 21^ Depressed individuals exhibit elevated levels of peripheral blood inflammatory biomarkers, most commonly C-reactive protein (CRP) and proinflammatory cytokines, as well as plasma, salivary and urinary cortisol.^22, 23, 24, 25, 26, 27^ As pregnancy *per se* is associated with increased maternal inflammation and HPA axis activity,^28, 29^ which can be further exacerbated by the experience of depression during this time,^30^ it has been hypothesized that inflammation and HPA axis dysregulation are likely candidate mechanisms for the biological embedding of maternal–fetal transmission of stress reactivity and vulnerability for affective psychopathology.^31, 32, 33, 34^ However, most studies detailing a link between maternal prenatal stress and offspring immune activation have been carried out in animals,^35, 36, 37^ with only one prospective human study demonstrating a link between maternal prenatal anxiety and reduced adaptive immunity in infancy,^38^ and a retrospective study detailing a link between maternal prenatal life events and elevated immune parameters in adult women.^39^ A greater number of studies have demonstrated an association between maternal prenatal stress and offspring HPA axis abnormalities,^8, 12, 40, 41^ with fewer studies examining HPA axis outcomes specifically in offspring of prenatally depressed mothers.^42^ Indeed, fetal exposure to synthetic maternal glucocorticoids has been shown to predict adolescent brain development and affective psychopathology.^43^

It is of particular note in this context that child maltreatment has also been identified as a prominent risk factor for subsequent mental and physical ill health,^44, 45, 46, 47, 48^ with individuals with a history of child maltreatment also exhibiting elevated inflammation and HPA axis abnormalities.^49, 50, 51, 52, 53^ Furthermore, recent clinical and epidemiological studies have described an association between exposure to maternal prenatal depression and an increased risk of the offspring being exposed to child maltreatment.^14, 54, 55, 56^ These findings suggest that prenatal depression and child maltreatment may be a part of the same putative pathway of vulnerability for stress-related disorders; however, because of the clinical overlap, it is currently unclear whether both kinds of stressors could lead to both inflammation and HPA axis abnormalities.

The aims of the current study were therefore to investigate (1) whether maternal prenatal depression predicts adulthood inflammation and HPA axis dysfunction in young adult offspring, and (2) whether offspring child maltreatment (and adulthood depression) moderate the effects of prenatal depression on these immune and neuroendocrine parameters. We sought to test these hypotheses using data recently collected through a new wave of assessment of offspring participants (aged 25) of the South London Child Development Study (SLCDS).

## Materials and methods

### Sample

The SLCDS is a prospective longitudinal UK birth cohort study that was setup in 1986.^13, 56, 57, 58, 59, 60^ All pregnant women who registered at one of two South London National Health Service general practices for antenatal care between 1 January 1986 and 31 December 1986 were invited to take part. One-to-one clinical interviews were carried out with expectant women at 20 and 36 weeks of pregnancy and 3 and 12 months postpartum, with offspring and mothers at 4, 11, 16 years and with offspring at 25 years. For this study, at 25 years the offspring were asked to provide blood and saliva samples, from which biomarkers of adulthood inflammation (high-sensitivity C-reactive protein (hs-CRP) and HPA axis activity (cortisol) were measured. At each phase of the study, independent researchers who were unaware of the content of previous assessments conducted the interviews.

**Figure 1:**
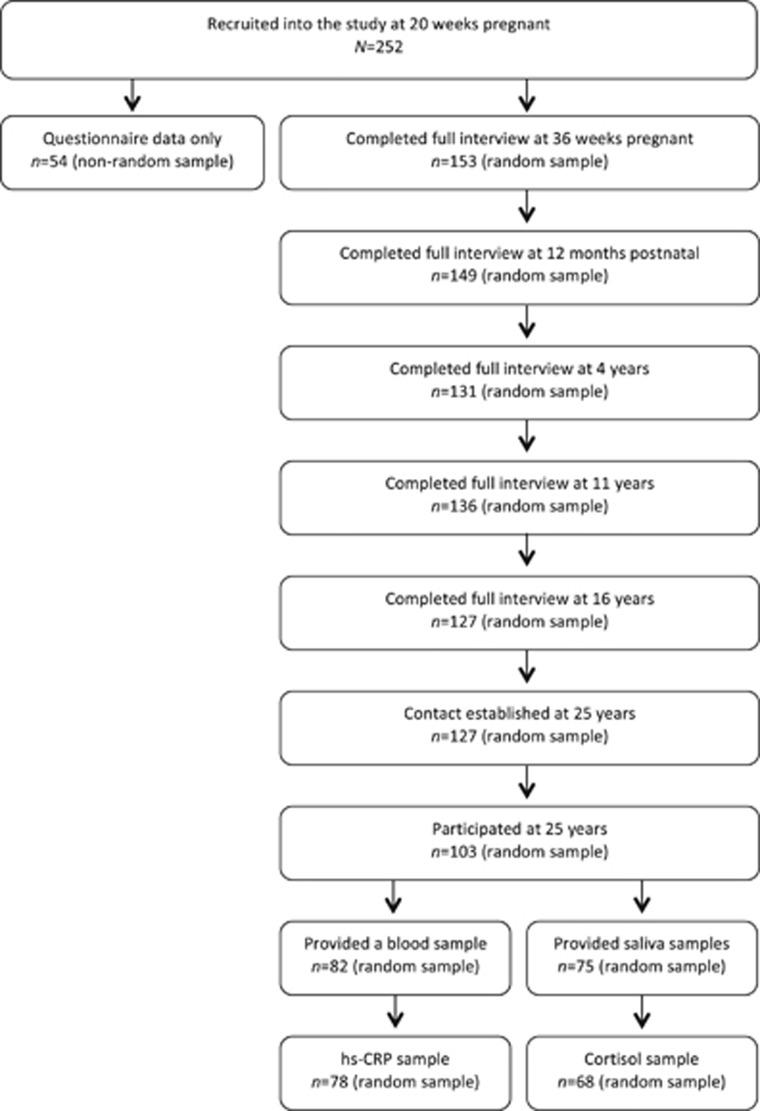
Flowchart of the study participation. hs-CRP, high-sensitivity C-reactive protein.

Two hundred and fifty-two women participated in the first assessment visit at 20 weeks' gestation. Because of time restraints, a 75% random subsample (hereon referred to as the ‘random sample') was selected for interview at 36 weeks pregnant and 3 months postnatal, with the remaining women completing postal self-report questionnaires only. The random sample did not differ statistically in any sociodemographic or clinical characteristics in comparison with the non-random sample,^57^ and is the sample of interest in this paper. Figure 1 depicts the progress of participation for the random sample from SLCDS onset to 25 years.

### Measures

#### Maternal prenatal depression

Maternal prenatal depression was assessed at 20 and 36 weeks of pregnancy using the Clinical Interview Schedule.^61^ International Classification of Diseases, Ninth Revision was used to make diagnoses of the women's current mental state (past 2 weeks). A dichotomous variable was created that detailed whether a mother had been clinically depressed at either time point in pregnancy (0=non-depressed and 1=depressed).

#### Offspring adulthood inflammation

Peripheral inflammation was indexed through the measurement of hs-CRP in the plasma. Blood samples were taken from the antecubital fossa using the BD Vacutainer Safety-Lok Blood Collection Set (BD, Oxford, UK). The participants were venipunctured in the early afternoon (*M*=13:34 h, s.d.=2.06) and were not fasted before sample collection. hs-CRP measurement was conducted blind to the participant status by King's College Hospital Pathology Department using the Siemens Advia 2400 (Siemens, Camberley, UK).

As CRP levels greater than 10.0 mg l^−1^ are indicative of acute inflammation, this upper limit was used as a cutoff to exclude individuals with potential acute inflammation from all further analyses;^51^ 4 of the 82 offspring who provided a blood sample were excluded from analysis on this basis, leaving an *n* of 78, which made up the ‘hs-CRP sample'. A continuous variable of offspring's hs-CRP levels (mg l^−1^) was generated. In addition, offspring were also dichotomized into clinically low and high inflammation using a cutoff of ⩾3.0 mg l^−1^, the American Heart Association definition for high cardiovascular disease risk.^62^

#### Offspring adulthood salivary cortisol

Offspring were instructed to collect four saliva samples in the first hour immediately after awakening ((i) upon awakening; (ii) +15 min; (iii) +30 min; (iv) +60 min) in order to assess the cortisol-awakening response (CAR). Offspring were instructed not to drink, eat, smoke or brush their teeth, and were given an instruction sheet and record log to detail the exact times of saliva sample collection. Saliva samples were assayed for cortisol using a standard commercial enzyme-linked immunosorbent assay (Salimetrics, Newmarket, UK). Cortisol values for each time point were used to estimate overall CAR values, using the trapezoid formula to calculate the area under the curve with respect to ground (AUC_G_).^63^ Seventy-five offspring returned their saliva samples; 7 offspring were excluded from analysis on the basis of (i) incomplete returned record sheets (*n*=4), (ii) illness on the day of sample collection (*n*=1), (iii) insufficient saliva to assay cortisol (*n*=1) and (iv) statistical outlier (*n*=1), leaving *n*=68 in the ‘cortisol sample'.

#### Offspring child maltreatment

Offspring exposure to child maltreatment (physical abuse, sexual abuse, emotional abuse and/or neglect up to age 17) was rated based on two independent assessment measures: the Childhood Experience of Care and Abuse Questionnaire (CECA.Q) conducted with offspring at 25 years and the Child and Adolescent Psychiatric Assessment (CAPA) conducted with offspring and primary caregiver (in most cases the mother) at 11 and 16 years.^64, 65, 66^ Physical and sexual abuse were rated based on offspring reports of severe incidents provided at 25 years using the CECA.Q (rated in accordance with cutoff guidelines published by Bifulco *et al.*),^65^ combined with the joint offspring and parental reports of severe instances of sexual and physical abuse provided at 11 and 16 years using the CAPA. For CAPA-recorded incidents, physical abuse was rated if participants reported severe incidents of abuse that involved at least some physical injury or force with potential for such, whereas sexual abuse was defined as severe incidents in which a perpetrator involved the offspring in activities for the perpetrator's own sexual gratification, such as fondling, oral contact, genital or anal intercourse. Emotional abuse and neglect were indexed through offspring ratings of severe parental antipathy and severe parental neglect up to 17 years using the CECA.Q in accordance with the rating guidelines published by Bifulco *et al.*^65^ A binary variable of maltreatment was rated if any one of the three types of severe abuse (physical, sexual and emotional) or severe neglect were ever present (0=non-maltreated and 1=severe maltreatment).

#### Offspring adulthood depression

Offspring depression (Diagnostic and Statistical Manual of Mental Disorders, 4th Edition (DSM-IV) diagnoses of major depressive disorder, depressive disorder not otherwise specified and dysthymic disorder) between ages 18 and 25 was assessed at 25 years using the Structured Clinical Interview for DSM-IV Axis I Disorders, Clinician Version.^67^ Diagnoses were rated in conjunction with the study psychiatrists (CMP and TC). A binary variable indicating a depressive disorder diagnosis was generated (0=non-depressed and 1=depressed).

#### Confounding variables

The following risk variables were included in analyses as potential confounders based on theoretical premise and previous studies:^42, 68^ offspring current body mass index (kg/m^2^); offspring current use of medication with potential effects on the immune system (contraceptive pill and injection, asthma pump, hormone regulation medication (for example, hair regrowth serums), antidepressants, non-steroidal anti-inflammatory drugs, corticosteroids, prescription anti-inflammatory drugs (0=no medication, 1=medication use);^51^ offspring current smoking (0, <5, 6–10, 11–15, 16–20 and 21+ cigarettes per day); offspring birth weight (g); offspring gestational age (whole weeks), offspring gender (0=male, 1=female), ethnicity (0=white British, 1=not white British); family social class (0=middle class, 1=working class).

### Ethics

Full ethical approval was obtained for all stages of the study at 25 years from London-Camberwell St Giles National Research Ethics Service Committee (reference number: 11/LO/0812). Written informed consent was obtained from all participants.

### Data analysis

First, we used hierarchical multiple regression models to test for association between maternal prenatal depression and offspring adulthood hs-CRP and cortisol. We then assessed whether child maltreatment and offspring adulthood depression contributed to the effects of prenatal depression on these biological outcomes using multiple regression and analyses of covariance (ANCOVAs). All statistical analyses were conducted in IBM SPSS Statistics Version 22 (IBM, Portsmouth, UK). The block-wise method of entry was used in all regression models, with the variable of interest entered at the final step. Multicollinearity was assessed using the variance inflation factor. All ANCOVAs and regression models were adjusted for potential confounding variables, and included only cases with a full data set.

## Results

### Descriptives

The mean hs-CRP value was 1.5 mg l^−1^ (s.d.=1.7, *n*=78); 13 (16.7%) offspring were categorized as having clinically high levels of inflammation (hs-CRP⩾3.0 mg l^−1^). The mean awakening cortisol value was 9.0 nmol l^−1^ (s.d.=4.0, *n*=68), and the mean CAR AUC_G_ value was 581.9 (s.d.=202.3, *n*=66). Cortisol levels rose during the first 15 min after awakening to a mean peak of 11.1 nmol l^−1^ (s.d.=4.4, *n*=68), decreasing slightly to a mean of 10.5 nmol l^−1^ (s.d.=4.2, *n*=68) at 30 min post awakening, and reducing further by 60 min post awakening to a mean of 7.9 nmol l^−1^ (s.d.=3.9, *n*=66). Inflammation was negatively correlated with CAR, as indexed by a significant negative correlation between hs-CRP values and elevation in cortisol levels at 30 min post awakening (*r*_s_=−0.3, *P*=0.02, *n*=58). Descriptive statistics summarizing sociodemographic and clinical characteristics of the hs-CRP and cortisol samples stratified by exposure to maternal prenatal depression are provided in Table 1.

### Maternal prenatal depression is associated with offspring adulthood inflammation

**Figure 2:**
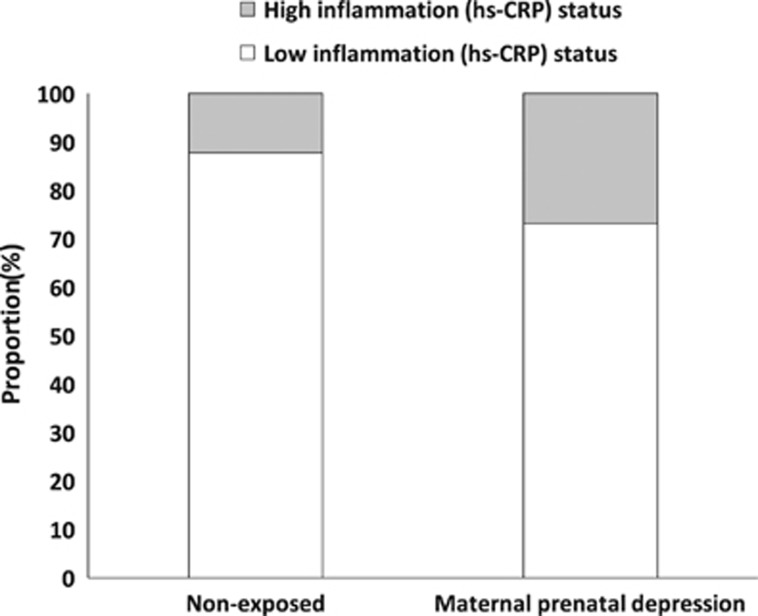
Proportion of offspring with clinically high versus low inflammation status as a function of exposure to maternal prenatal depression. hs-CRP, high-sensitivity C-reactive protein.

We tested this hypothesis using hierarchical multiple regression models. In a hierarchical multiple linear regression model (controlling for gender, ethnicity, family social class, gestational age, birth weight, adulthood depression, child maltreatment, smoking, medication use and body mass index), maternal prenatal depression predicted significantly elevated offspring hs-CRP levels (*B*=1.1, *t*=2.9, *P*=0.005, 95% confidence interval (CI) (0.3, 1.8), model *R*^2^=.45, F_11,63_=4.6, *P*<0.001, *n*=75; see Supplementary Figure 1 for scatterplot). Similarly, an adjusted hierarchical multiple logistic regression model revealed a significant association between maternal prenatal depression and offspring classified with clinically high inflammation based on the dichotomized hs-CRP values, with more than an 11-fold increased risk in exposed offspring (Wald) statistic=4.2, *P*=0.041, odds ratio=11.8, 95% CI (1.1, 127.0), model *χ*^*2*^_1_=32.3, degree of freedom=11, *P*=0.001, *n*=75). Notably, neither child maltreatment nor adulthood depression predicted elevated overall hs-CRP levels or dichotomized hs-CRP levels. Full model statistics are summarized in Table 2, and a graphical representation of offspring-dichotomized hs-CRP levels as a function of exposure to maternal prenatal depression is presented in Figure 2. Next, we tested whether child maltreatment and adulthood depression moderated the effects of maternal prenatal depression on offspring adulthood inflammation. Moderation analyses did not reveal any evidence of statistically significant interactions between child maltreatment and maternal prenatal depression, or adulthood depression and prenatal maternal depression, for either overall or dichotomized hs-CRP levels.

**Figure 3:**
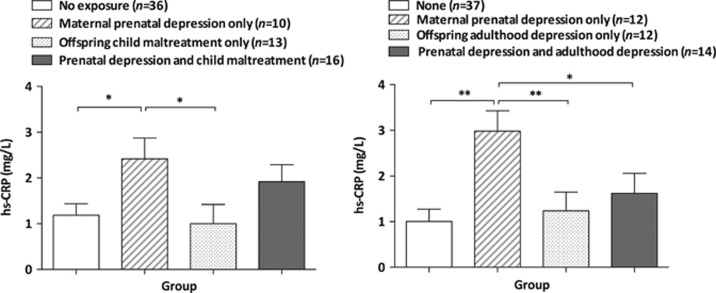
Estimates of offspring mean hs-CRP levels as a function of exposure to maternal prenatal depression, offspring child maltreatment and depression in adulthood, adjusted for covariates (*P<0.05, **P<0.01). hs-CRP, high-sensitivity C-reactive protein.

Finally, supplementary ANCOVAs were conducted to examine whether exposure to maternal prenatal depression, child maltreatment and adulthood depression exert differential or cumulative effects on hs-CRP levels, see Figure 3. In the first ANCOVA we compared the following four groups of offspring: (i) not exposed to either prenatal maternal depression or child maltreatment (*n*=36); (ii) exposed to only prenatal maternal depression (*n*=10); (iii) exposed to only child maltreatment (*n*=13); (iv) exposed to both prenatal maternal depression and child maltreatment (*n*=16). The ANCOVA (adjusted for all covariates) revealed a significant main effect (F_3,62_=2.8, *P*=0.047, *n*=75), with *post hoc* analyses revealing that offspring exposed to only maternal prenatal depression had significantly higher hs-CRP values in comparison with non-exposed offspring (estimated mean difference=1.2, s.e.=0.5, *P*=0.02), and offspring exposed to only child maltreatment (estimated mean difference=1.4, s.e.=0.6, *P*=0.03), with no difference between those exposed to only child maltreatment and non-exposed offspring (estimated mean difference=−0.2 s.e.=0.5, *P*=0.7) and no further increase in offspring exposed to both insults compared with offspring exposed to only maternal prenatal depression (estimated mean difference=0.5 s.e.=0.6, *P*=0.4). Using a similar approach, a second ANCOVA (adjusted for all covariates; F_3,62_=4.5, *P*=0.006, *n*=75) revealed that offspring exposed to only maternal prenatal depression (*n*=12) had significantly higher hs-CRP values in comparison with non-exposed and non-depressed offspring (*n*=37; estimated mean difference=1.8, s.e.=0.5, *P*=0.001), with depressed-only offspring (*n*=12; estimated mean difference=1.5, s.e.=0.6, *P*=0.009) and with exposed and depressed offspring (*n*=14; estimated mean difference=1.4, s.e.=0.6, *P*=0.02). We did not observe any significant differences between depressed-only offspring and non-exposed offspring (estimated mean difference=0.2, s.e.=0.5, *P*=0.6).

### Child maltreatment is associated with changes in offspring adulthood CAR

Hierarchical multiple linear regression models (controlling for gender, ethnicity, family social class, gestational age, birth weight, adulthood depression, child maltreatment, smoking, medication use and body mass index) revealed no significant association between maternal prenatal depression and offspring raw cortisol levels or CAR (awakening: *B*=0.4, *P*=0.8, *n*=65; +15 min: *B*=−0.3, *P*=0.8, *n*=65; +30 min: *B*=−0.4, *P*=0.8, *n*=65; +60 min: *B*=−1.0, *P*=0.4, *n*=63; AUC_G_: *B*=−22.5, *P*=0.7, *n*=65). In contrast, offspring child maltreatment predicted a significantly elevated CAR (AUC_G_: *B*=161.9, *t*=2.8, *P*=0.007, 95% CI (45.4, 278.4), model *R*^2^=0.31, F_11,52_=2.1, *P*=0.03, *n*=63; see Supplementary Figure 2 for scatterplot). Moderation analyses did not reveal any evidence of statistically significant interactions between either child maltreatment and maternal prenatal depression, or adulthood depression and prenatal maternal depression, on cortisol levels.

To investigate further whether exposure to child maltreatment and experiencing depression in adulthood exert a cumulative effect on the CAR, we compared the following four groups of offspring: (i) not exposed to child maltreatment and not depressed in adulthood (*n*=34); (ii) exposed to child maltreatment only (*n*=12); (iii) depressed in adulthood only (*n*=6); (iv) exposed to child maltreatment and depressed in adulthood (*n*=14). ANCOVA revealed a significant main effect on CAR AUC_G_ levels (F_3,52_=5.4, *P*=0.003, *n*=64). *Post hoc* comparisons revealed that offspring exposed to only child maltreatment had significantly higher values in comparison with non-maltreated and non-depressed offspring (estimated mean difference=251.6, s.e.=65.2, *P*<0.001), and maltreated and depressed offspring (estimated mean difference=244.7, s.e.=74.5, *P*=0.002).

## Discussion

In the present study we use a 26-year prospective longitudinal design to demonstrate, for we believe the first time, that exposure to maternal prenatal depression predicts elevated offspring inflammation in early adulthood. We find that this effect is not accounted for by the experience of subsequent child maltreatment or depression in adulthood. Rather, our data suggest that maternal prenatal depression has a persistent effect on offspring inflammation at the age of 25 that is independent of subsequent adverse experiences. Interestingly, we do not find support for our hypothesis of an effect of maternal prenatal depression on offspring 25-year awakening cortisol levels; however, our data indicate that offspring experience of child maltreatment predicts an elevated adulthood CAR.

### Inflammation

Our finding that maternal prenatal depression predicts inflammation at 25 years extends recent animal and human work that reports a link between prenatal stress exposures and altered immune parameters in the infancy period and in women.^35, 36, 37, 38, 39^ It thereby provides some of the strongest evidence to date that prenatal adversities, specifically maternal depression during pregnancy, have persistent and independent effects on the offspring's inflammatory system activation during early adulthood. Adult offspring whose mothers were clinically depressed during pregnancy were not only significantly more likely to have greater hs-CRP levels than offspring of non-prenatally depressed mothers, but also were more likely to have inflammation reaching a clinically significant threshold, which is itself a risk for subsequent chronic metabolic conditions such as cardiovascular disease.^69, 70^ These data thereby add to the literature on the developmental origins of health and disease risk.

It is interesting that our data indicate that there was no moderating effect of child maltreatment on the association between maternal prenatal depression and offspring hs-CRP. Furthermore, our results indicate that offspring with a history of only child maltreatment do not show elevated hs-CRP levels in the context of measurement of maternal prenatal depression. This may seem in contradiction with studies (including from this group), showing that adults with a history of child maltreatment have elevated inflammation.^51, 52, 53, 71^ Nevertheless, given that child maltreatment has been shown to be predicted by maternal prenatal depression,^14, 54^ and that previous studies have measured only child maltreatment but not prenatal depression,^51, 52, 53, 71^ it is plausible that the ‘unmeasured' effects of maternal prenatal depression in these studies may have been attributed to child maltreatment in a manner reflecting residual confounding.^68^

This account thereby advocates that prenatal stress has modulatory effects on inflammatory system development.^6^ Indeed, studies in rodents have demonstrated that pups of prenatally stressed dams show elevated levels of circulating proinflammatory cytokines^72^ and cytokine mRNA expression.^73, 74^ There are also reports of stress-induced activation of immune pathways within the placenta, including increased gene expression for interleukin-6 and interleukin-1 beta.^75^ Such gene-expression changes may be underpinned by epigenetic effects, which are particularly plausible putative mechanisms for long-term effects. Nevertheless, given that both the inflammatory system and the HPA axis are hyperactive in depressed pregnant women, another potential (non-exclusive) mechanism could be the indirect chronic activation of the offspring immune system via changes in HPA axis function.^28, 29, 30^ For example, a recent study reported HPA axis hyperactivity and glucocorticoid resistance, in the context of increased cytokine levels, in pregnant women with high sociodemographic risks.^76^

### HPA axis

The fact that we do not find an association between maternal prenatal depression and offspring adulthood cortisol is interesting, given that a link has been demonstrated with diurnal cortisol measured at the age of 15 in a different sample.^42^ One potential explanation for these differences could be attributed to methodological factors. The aforementioned study comprised a large sample, yet with only small effect sizes being observed,^42^ and therefore it is possible that our design lacked the power to detect a potential adulthood effect. Furthermore, we measured the CAR, which reflects different aspects of HPA axis functioning compared with diurnal cortisol.^77, 78^ However, it is also possible that the effects of prenatal depression on HPA axis function are present in adolescence, but do not persist into adulthood. Of note, our finding that child maltreatment predicts an altered cortisol profile is consistent with the literature linking childhood adversity with HPA axis dysfunction.^50^

### Strengths and limitations

Alongside the numerous strengths of this study, such as the use of a prospective design starting in pregnancy through 26 years to chart offspring development, and the prospective collection of data through one-to-one interviews, there are limitations that need to be highlighted. First, the SLCDS is drawn from an urban, predominantly working class population of families of white ethnic origin; thus, these results may not be representative of the wider population. Second, the majority of mothers were diagnosed with International Classification of Diseases, Ninth Revision neurotic depression during pregnancy, a diagnosis in which anxiety can also be present along with depressed mood;^79^ therefore, it is possible that our observed effects could be attributed to anxiety, or mixed anxiety and depression, rather than depression alone. Third, only one index of inflammation (hs-CRP) and HPA axis function (awakening cortisol) was measured. Fourth, because of the high levels of clinical overlap between risk and psychopathology constructs, group sizes were heavily uneven when making group comparisons (albeit statistical corrections were applied to account for this).

### Implications and conclusions

Our findings suggest that maternal prenatal depression has persistent effects on offspring inflammation that are observable up to 25 years after birth. We do not find an effect of prenatal maternal depression on offspring adulthood HPA axis activity, suggesting a specific effect on programming inflammation. Findings are consistent with the developmental origins of health and disease hypothesis of the biological embedding of gestational psychosocial adversity into vulnerability for future physical and mental illness. Within a preventative medicine framework, pregnancy is an opportune time to intervene, given women's high contact with clinical services, in order to protect against high levels of further physical and mental illness in the next generation of adults.

## Figures and Tables

**Table 1:** Group differences between prenatally exposed versus non-exposed offspring for hs-CRP and cortisol samples

	*hs-CRP sample (*n=*78)*			*Cortisol sample (*n=*68)*		
	*Exposure to prenatal maternal depression*		*Group effect (*P)	*Exposure to prenatal maternal depression*		*Group effect (*P)
	*None (*n=*50, 64.1%)*	*Exposed (*n=*28, 35.9%)*		*None (*n=*46, 67.6%)*	*Exposed (*n=*22, 32.4%)*	
*Basic characteristics*						
Ethnicity, % white British	72.0	64.3	0.48	73.9	59.1	0.22
Gender, % male	54.0	41.0	0.35	54.3	45.5	0.49
Family social class, % middle class	16.0	14.3	0.84	15.2	13.6	0.86
						
*Perinatal factors*						
Birth weight (g), *M* (s.d.)	3408.3 (479.6)	3344.4 (465.2)	0.53	3355.5 (445.9)	3436.7 (495.0)	0.69
Gestational age (weeks), *M* (s.d.)	39.9 (1.5)	39.8 (1.9)	0.80	39.9 (1.6)	40.0 (1.7)	0.98
						
*Clinical factors*						
Child maltreatment, % exposed	26.0	57.1	0.006	28.3	59.1	0.01
Adulthood depression, % depressed	24.0	53.6	0.008	19.6	54.5	0.003
						
*Health factors*						
Currently smoke, %	40.8	38.5	0.84^a^	34.8	30.0	0.71^b^
Currently using medication, %	30.0	25.0	0.64	34.8	27.3	0.54
Adulthood BMI, *M (s.d.)*	25.4 (6.0)	25.6 (4.0)	0.56	26.6 (7.0)	23.7 (3.4)	0.15

Abbreviations: BMI, body mass index; hs-CRP, high-sensitivity C-reactive protein. The independent samples *t-*test was used for group comparisons comprising continuous parametric data, whereas the Mann–Whitney test was applied to non-parametric continuous data. Pearson's *χ*^2^-test for independence was used for the analysis of categorical data.

a *n*=75.

b *n*=66.

**Table 2:** Hierarchical multiple linear and logistic regression models of hs-CRP levels and clinically high inflammation status

*Predictors*	*Overall hs-CRP levels*			*Dichotomized (high versus low) hs-CRP*	
	R^*2*^	B *(s.e.)*	*95% CI for* B	*Exp(*B)	*95% CI for Exp(*B)
*Block 1*	*0.06*				
Gender		−0.02 (0.4)	−0.6, 0.7	0.9	0.1, 6.1
Family social class		−0.5 (0.5)	−1.5, 0.4	0.2	0.01, 2.7
Ethnicity		−0.6 (0.3)	−1.3, 1.6	0.1	0.01, 1.0
					
*Block 2*	*0.07*				
Adulthood depression		−0.4 (0.4)	1.3, 0.3	1.0	0.1, 10.2
Child maltreatment		−0.3 (0.4)	−1.1, 0.5	0.1	0.01, 2.7
					
*Block 3*	*0.35*				
BMI		0.1 (0.03)^***^	0.1, 0.2	1.3^**^	1.1, 1.6
Medication use		1.6 (0.4)^**^	0.8, 2.5	51.1^**^	3.2, 819.3
Smoking		0.1 (0.1)	−0.2, 0.3	0.08	0.3, 2.2
					
*Block 4*	*0.37*				
Gestational age		−0.1 (0.1)	−0.3, 0.2	0.6	0.3, 1.4
Birth weight		0.01 (0.01)	−0.01, 0.01	1.0	1.0, 1.1
					
*Block 5*	*0.45*				
Maternal prenatal depression		1.1 (0.4)^**^	0.3, 1.8	11.8*	1.1, 127.0

Abbreviations: BMI, body mass index; CI, confidence interval; hs-CRP, high-sensitivity C-reactive protein.

*n*=75. *P<0.05, ^**^P<0.01, ^***^P<0.001.

## References

[bib1] Talge NM, Neal C, Glover V. Antenatal maternal stress and long-term effects on child neurodevelopment: how and why? J Child Psychol Psychiatry 2007; 48: 245–261. 17355398 10.1111/j.1469-7610.2006.01714.x PMC11016282

[bib2] Glover V. Annual research review: prenatal stress and the origins of psychopathology: an evolutionary perspective. J Child Psychol Psychiatry 2011; 52: 356–367. 21250994 10.1111/j.1469-7610.2011.02371.x

[bib3] Van den Bergh BRH, Van Calster B, Smits T, Van Huffel S, Lagae L. Antenatal maternal anxiety is related to HPA-axis dysregulation and self-reported depressive symptoms in adolescence: a prospective study on the fetal origins of depressed mood. Neuropsychopharmacology 2008; 33: 536–545. 17507916 10.1038/sj.npp.1301450

[bib4] Sandman CA, Buss C, Head K, Davis EP. Fetal Exposure to maternal depressive symptoms is associated with cortical thickness in late childhood. Biol Psychiatry 2014; 15: 324–334. 10.1016/j.biopsych.2014.06.025 PMC4289467 25129235

[bib5] Rifkin-Graboi A, Bai J, Chen H, Hameed WB, Sim LW, Tint MT et al. Prenatal maternal depression associates with microstructure of right amygdala in neonates at birth. Biol Psychiatry 2013; 74: 837–844. 23968960 10.1016/j.biopsych.2013.06.019

[bib6] Entringer S, Buss C, Swanson JM, Copper DM, Wing DA, Waffarn F et al. Fetal programming of body composition, obesity, and metabolic function: the role of intrauterine stress and stress biology. J Nutr Metab 2012; 2012: 1–16. 10.1155/2012/632548 PMC3359710 22655178

[bib7] Van Dijk AE, van Eijsden M, Stronks K, Gemke RJBJ, Vrijkotte TGM. The association between prenatal psychosocial stress and blood pressure in the child at age 5-7 years. PLoS One 2012; 7: e43548. 22927987 10.1371/journal.pone.0043548 PMC3424234

[bib8] Charil A, Laplante DP, Vaillancourt C, King S. Prenatal stress and brain development. Brain Res Rev 2010; 65: 56–79. 20550950 10.1016/j.brainresrev.2010.06.002

[bib9] Barker D. Fetal origins of coronary heart disease. Br Med J 1995; 311: 171–174. 7613432 10.1136/bmj.311.6998.171 PMC2550226

[bib10] Barker D, Hales CN, Fall CH, Osmond C, Phipps K, Clark PM. Type 2 (non-insulin-dependent) diabetes mellitus, hypertension and hyperlipidaemia (syndrome X): relation to reduced fetal growth. Diabetologia 1993; 36: 62–67. 8436255 10.1007/BF00399095

[bib11] Barker D, Clark P. Fetal undernutrition and disease in later life. Rev Reprod 1997; 2: 105–112. 9414472 10.1530/ror.0.0020105

[bib12] Reynolds RM. Glucocorticoid excess and the developmental origins of disease: two decades of testing the hypothesis. Psychoneuroendocrinology 2013; 38: 1–11. 22998948 10.1016/j.psyneuen.2012.08.012

[bib13] Pawlby S, Hay DF, Sharp D, Waters CS, O'Keane V. Antenatal depression predicts depression in adolescent offspring: prospective longitudinal community-based study. J Affect Disord 2009; 113: 236–243. 18602698 10.1016/j.jad.2008.05.018

[bib14] Plant DT, Pariante CM, Sharp D, Pawlby S. Maternal depression during pregnancy and offspring depression in adulthood: role of child maltreatment. Br J Psychiatry 2015; 207: 213–220. 26045352 10.1192/bjp.bp.114.156620 PMC4555443

[bib15] Pearson RM, Evans J, Kounali D, Lewis G, Heron J, Ramchandani PG et al. Maternal depression during pregnancy and the postnatal period: risks and possible mechanisms for offspring depression at age 18 years. JAMA Psychiatry 2013; 70: 1312–1319. 24108418 10.1001/jamapsychiatry.2013.2163 PMC3930009

[bib16] Pariante C, Lightman SL. The HPA axis in major depression: classical theories and new developments. Trends Neurosci 2008; 31: 464–468. 18675469 10.1016/j.tins.2008.06.006

[bib17] Khandaker GM, Pearson RM, Zammit S, Lewis G, Jones PB. Association of serum interleukin 6 and C-reactive protein in childhood with depression and psychosis in young adult life: a population-based longitudinal study. JAMA Psychiatry 2014; 71: 1121–1128. 25133871 10.1001/jamapsychiatry.2014.1332 PMC4561502

[bib18] Holsboer F. The corticosteroid receptor hypothesis of depression. Neuropsychopharmacology 2000; 23: 477–501. 11027914 10.1016/S0893-133X(00)00159-7

[bib19] Raison CL, Capuron L, Miller AH. Cytokines sing the blues: inflammation and the pathogenesis of depression. Trends Immunol 2006; 27: 24–31. 16316783 10.1016/j.it.2005.11.006 PMC3392963

[bib20] Miller A, Maletic V, Raison CL. Inflammation and its discontents: the role of cytokines in the pathophysiology of major depression. Biol Psychiatry 2009; 65: 732–741. 19150053 10.1016/j.biopsych.2008.11.029 PMC2680424

[bib21] Zunszain PA, Anacker C, Cattaneo A, Carvalho LA, Pariante CM. Glucocorticoids, cytokines and brain abnormalities in depression. Prog Neuropsychopharmacol Biol Psychiatry 2011; 35: 722–729. 20406665 10.1016/j.pnpbp.2010.04.011 PMC3513408

[bib22] Dowlati Y, Herrmann N, Swardfager W, Liu H, Sham L, Reim EK et al. A meta-analysis of cytokines in major depression. Biol Psychiatry 2010; 67: 446–457. 20015486 10.1016/j.biopsych.2009.09.033

[bib23] Liu Y, Ho RC-M, Mak A. Interleukin (IL)-6, tumour necrosis factor alpha (TNF-α) and soluble interleukin-2 receptors (sIL-2R) are elevated in patients with major depressive disorder: a meta-analysis and meta-regression. J Affect Disord 2012; 139: 230–239. 21872339 10.1016/j.jad.2011.08.003

[bib24] Zorrilla EP, Luborsky L, McKay JR, Rosenthal R, Houldin A, Tax A et al. The relationship of depression and stressors to immunological assays: a meta-analytic review. Brain Behav Immun 2001; 15: 199–226. 11566046 10.1006/brbi.2000.0597

[bib25] Nemeroff CB, Vale WW. The neurobiology of depression: inroads to treatment and new drug discovery. J Clin Psychiatry 2005; 66: 5–13. 16124836

[bib26] Bhagwagar Z, Hafizi S, Cowen PJ. Increased salivary cortisol after waking in depression. Psychopharmacology 2005; 182: 54–57. 15991000 10.1007/s00213-005-0062-z

[bib27] Jarcho MR, Slavich GM, Tylova-Stein H, Wolkowitz OM, Burke HM. Dysregulated diurnal cortisol pattern is associated with glucocorticoid resistance in women with major depressive disorder. Biol Psychol 2013; 93: 150–158. 23410758 10.1016/j.biopsycho.2013.01.018 PMC3687535

[bib28] Coussons-Read ME, Okun ML, Schmitt MP, Giese S. Prenatal stress alters cytokine levels in a manner that may endanger human pregnancy. Psychosom Med 2005; 67: 625–631. 16046378 10.1097/01.psy.0000170331.74960.ad

[bib29] Coussons-Read ME, Okun ML, Nettles CD. Psychosocial stress increases inflammatory markers and alters cytokine production across pregnancy. Brain Behav Immun 2007; 21: 343–350. 17029703 10.1016/j.bbi.2006.08.006

[bib30] O'Keane V, Lightman S, Marsh M, Pawlby S, Papadopoulos AS, Taylor A et al. Increased pituitary-adrenal activation and shortened gestation in a sample of depressed pregnant women: a pilot study. J Affect Disord 2011; 130: 300–305. 21093926 10.1016/j.jad.2010.10.004

[bib31] Miller G, Chen E, Sze J, Marin T, Arevalo JMG, Doll R et al. A functional genomic fingerprint of chronic stress in humans: blunted glucocorticoid and increased NF-kappaB signaling. Biol Psychiatry 2008; 64: 266–272. 18440494 10.1016/j.biopsych.2008.03.017 PMC2581622

[bib32] Miller G, Chen E, Zhou ES. If it goes up, must it come down? Chronic stress and the hypothalamic-pituitary-adrenocortical axis in humans. Psychol Bull 2007; 133: 25–45. 17201569 10.1037/0033-2909.133.1.25

[bib33] Miller A. Norman cousins lecture: mechanisms of cytokine-induced behavioral changes: psychoneuroimmunology at the translational interface. Brain Behav Immun 2009; 23: 149–158. 18793712 10.1016/j.bbi.2008.08.006 PMC2745948

[bib34] Horowitz M, Zunszain P, Anacker C, Musaelyan K, Pariante C Glucocorticoids and Inflammation; a double-headed sword in depression? How do neuroendocrine and inflammatory pathways interact during stress to contribute to the pathogenesis of depression? In: Halaris A, Leonard B eds Inflammation in Psychiatry. Karger Publishers: Basel, 2013; 127–143. 10.1159/000343980 25224896

[bib35] Diz-Chaves Y, Astiz M, Bellini MJ, Garcia-Segura LM. Prenatal stress increases the expression of proinflammatory cytokines and exacerbates the inflammatory response to LPS in the hippocampal formation of adult male mice. Brain Behav Immun 2013; 28: 196–206. 23207108 10.1016/j.bbi.2012.11.013

[bib36] Coe CL. Prenatal stress diminishes the cytokine response of leukocytes to endotoxin stimulation in juvenile rhesus monkeys. J Clin Endocrinol Metab 2002; 87: 675–681. 11836303 10.1210/jcem.87.2.8233

[bib37] Couret D, Jamin A, Kuntz-Simon G, Prunier A, Merlot E. Maternal stress during late gestation has moderate but long-lasting effects on the immune system of the piglets. Vet Immunol Immunopathol 2009; 131: 17–24. 19362376 10.1016/j.vetimm.2009.03.003

[bib38] O'Connor TG, Winter MA, Hunn J, Carnahan J, Pressman EK, Glover V et al. Prenatal maternal anxiety predicts reduced adaptive immunity in infants. Brain Behav Immun 2013; 32: 21–28. 23439080 10.1016/j.bbi.2013.02.002 PMC3686987

[bib39] Entringer S, Kumsta R, Nelson EL, Hellhammer DH, Wadhwa PD, Wüst S. Influence of prenatal psychosocial stress on cytokine production in adult women. Dev Psychobiol 2008; 50: 579–587. 18683180 10.1002/dev.20316 PMC2838479

[bib40] Weinstock M. The long-term behavioural consequences of prenatal stress. Neurosci Biobehav Rev 2008; 32: 1073–1086. 18423592 10.1016/j.neubiorev.2008.03.002

[bib41] Seckl JR, Holmes MC. Mechanisms of disease: glucocorticoids, their placental metabolism and fetal “programming” of adult pathophysiology. Nat Clin Pract Endocrinol Metab 2007; 3: 479–488. 17515892 10.1038/ncpendmet0515

[bib42] O'Donnell KJ, Glover V, Jenkins J, Browne D, Ben-Shlomo Y, Golding J et al. Prenatal maternal mood is associated with altered diurnal cortisol in adolescence. Psychoneuroendocrinology 2013; 38: 1630–1638. 23433748 10.1016/j.psyneuen.2013.01.008 PMC3695029

[bib43] Davis EP, Sandman CA, Buss C, Wing DA, Head K. Fetal glucocorticoid exposure is associated with preadolescent brain development. Biol Psychiatry 2013; 74: 647–655. 23611262 10.1016/j.biopsych.2013.03.009 PMC3985475

[bib44] Bifulco A, Moran PM, Baines R, Bunn A, Stanford K. Exploring psychological abuse in childhood: II. Association with other abuse and adult clinical depression. Bull Menninger Clin 2002; 66: 241–258. 12448629 10.1521/bumc.66.3.241.23366

[bib45] Nanni V, Uher R, Danese A. Childhood maltreatment predicts unfavorable course of illness and treatment outcome in depression: a meta-analysis. Am J Psychiatry 2012; 169: 141–151. 22420036 10.1176/appi.ajp.2011.11020335

[bib46] Widom C, DuMont K, Czaja SJ. A prospective investigation of major depressive disorder and comorbidity in abused and neglected children grown up. Arch Gen Psychiatry 2007; 64: 49–56. 17199054 10.1001/archpsyc.64.1.49

[bib47] Norman RE, Byambaa M, De R, Butchart A, Scott J, Vos T. The long-term health consequences of child physical abuse, emotional abuse, and neglect: a systematic review and meta-analysis. PLoS Med 2012; 9: e1001349. 23209385 10.1371/journal.pmed.1001349 PMC3507962

[bib48] Green JG, McLaughlin KA, Berglund PA, Gruber MJ, Sampson NA, Zaslavsky AM et al. Childhood adversities and adult psychiatric disorders in the national comorbidity survey replication I: associations with first onset of DSM-IV disorders. Arch Gen Psychiatry 2010; 67: 113–123. 20124111 10.1001/archgenpsychiatry.2009.186 PMC2822662

[bib49] Heim C, Newport DJ, Mletzko T, Miller AH, Nemeroff CB. The link between childhood trauma and depression: insights from HPA axis studies in humans. Psychoneuroendocrinology 2008; 33: 693–710. 18602762 10.1016/j.psyneuen.2008.03.008

[bib50] Nemeroff CB. Neurobiological consequences of childhood trauma. J Clin Psychiatry 2004; 65: 18–28. 14728093

[bib51] Danese A, Pariante CM, Caspi A, Taylor A, Poulton R. Childhood maltreatment predicts adult inflammation in a life-course study. Proc Natl Acad Sci USA 2007; 104: 1319–1324. 17229839 10.1073/pnas.0610362104 PMC1783123

[bib52] Slopen N, Lewis TT, Gruenewald TL, Mujahid MS, Ryff CD, Albert MA et al. Early life adversity and inflammation in African Americans and whites in the midlife in the United States survey. Psychosom Med 2010; 72: 694–701. 20595419 10.1097/PSY.0b013e3181e9c16f PMC2939196

[bib53] Coelho R, Viola TW, Walss-Bass C, Brietzke E, Grassi-Oliveira R. Childhood maltreatment and inflammatory markers: a systematic review. Acta Psychiatr Scand 2014; 129: 180–192. 24205846 10.1111/acps.12217

[bib54] Lereya ST, Wolke D. Prenatal family adversity and maternal mental health and vulnerability to peer victimisation at school. J Child Psychol Psychiatry 2013; 54: 644–652. 23121554 10.1111/jcpp.12012

[bib55] Pariante CM. Depression during pregnancy: molecular regulations of mothers' and children's behaviour. Biochem Soc Trans 2014; 42: 582–586. 24646281 10.1042/BST20130246

[bib56] Pawlby S, Hay D, Sharp D, Waters CS, Pariante CM. Antenatal depression and offspring psychopathology: the influence of childhood maltreatment. Br J Psychiatry 2011; 199: 106–112. 21727235 10.1192/bjp.bp.110.087734

[bib57] Sharp D. Childbirth Related Emotional Disorders In Primary Care: A Longitudinal Prospective Study. University of London: London, UK, 1992.

[bib58] Hay DF, Pawlby S, Waters CS, Perra O, Sharp D. Mothers' antenatal depression and their children's antisocial outcomes. Child Dev 2010; 81: 149–165. 20331659 10.1111/j.1467-8624.2009.01386.x

[bib59] Plant DT, Barker ED, Waters CS, Pawlby S, Pariante CM. Intergenerational transmission of maltreatment and psychopathology: the role of antenatal depression. Psychol Med 2013; 43: 519–528. 22694795 10.1017/S0033291712001298 PMC3558981

[bib60] Bauer A, Pawlby S, Plant DT, King D, Pariante CM, Knapp M. Perinatal depression and child development: exploring the economic consequences from a South London cohort. Psychol Med 2015; 45: 51–61. 25066467 10.1017/S0033291714001044 PMC4341975

[bib61] Goldberg DP, Cooper B, Eastwood MR, Kedward HB, Shepherd M. A standardized psychiatric interview for use in community surveys. Br J Prev Soc Med 1970; 24: 18–23. 5435083 10.1136/jech.24.1.18 PMC1059220

[bib62] Ridker P. Clinical application of c-reactive protein for cardiovascular disease detection and prevention. Circulation 2003; 107: 363–369. 12551853 10.1161/01.cir.0000053730.47739.3c

[bib63] Pruessner J, Kirschbaum C, Meinlschmid G, Hellhammer DH. Two formulas for computation of the area under the curve represent measures of total hormone concentration versus time-dependent change. Psychoneuroendocrinology 2003; 28: 916–931. 12892658 10.1016/s0306-4530(02)00108-7

[bib64] Smith N, Lam D, Bifulco A, Checkley S. Childhood experience of care and abuse questionnaire (CECA.Q). Validation of a screening instrument for childhood adversity in clinical populations. Soc Psychiatry Psychiatr Epidemiol 2002; 37: 572–579. 12545234 10.1007/s00127-002-0589-9

[bib65] Bifulco A, Bernazzani O, Moran PM, Jacobs C. The childhood experience of care and abuse questionnaire (CECA.Q): validation in a community series. Br J Clin Psychol 2005; 44: 563–581. 16368034 10.1348/014466505X35344

[bib66] Angold A, Costello EJ. The child and adolescent psychiatric assessment (CAPA). J Am Acad Child Adolesc Psychiatry 2000; 39: 39–48. 10638066 10.1097/00004583-200001000-00015

[bib67] First MB, Spitzer RL, Gibbon M, Williams JB. Structured Clinical Interview for DSM-IV Axis I Disorders, Clinician Version (SCID-CV). American Psychiatric Press: Washington, USA, 1996.

[bib68] Thapar A, Rutter M. Do prenatal risk factors cause psychiatric disorder? Be wary of causal claims. Br J Psychiatry 2009; 195: 100–101. 19648537 10.1192/bjp.bp.109.062828

[bib69] Pearson TA, Mensah GA, Alexander RW, Anderson JL, Cannon RO, Criqui M et al. Markers of inflammation and cardiovascular disease: application to clinical and public health practice: a statement for healthcare professionals from the Centers for Disease Control and Prevention and the American Heart Association. Circulation 2003; 107: 499–511. 12551878 10.1161/01.cir.0000052939.59093.45

[bib70] Hansson GK. Inflammation, atherosclerosis, and coronary artery disease. N Engl J Med 2005; 352: 1685–1695. 15843671 10.1056/NEJMra043430

[bib71] Carpenter LL, Gawuga CE, Tyrka AR, Lee JK, Anderson GM, Price LH. Association between plasma IL-6 response to acute stress and early-life adversity in healthy adults. Neuropsychopharmacology 2010; 35: 2617–2623. 20881945 10.1038/npp.2010.159 PMC2978751

[bib72] Beijers R, Buitelaar JK, de Weerth C. Mechanisms underlying the effects of prenatal psychosocial stress on child outcomes: beyond the HPA axis. Eur Child Adolesc Psychiatry 2014; 23: 943–956. 24875898 10.1007/s00787-014-0566-3

[bib73] Szczesny E, Basta-Kaim A, Slusarczyk J, Trojan E, Glombik K, Regulska M et al. The impact of prenatal stress on insulin-like growth factor-1 and pro-inflammatory cytokine expression in the brains of adult male rats: the possible role of suppressors of cytokine signaling proteins. J Neuroimmunol 2014; 276: 37–46. 25151093 10.1016/j.jneuroim.2014.08.001

[bib74] Vanbesien-Mailliot CCA, Wolowczuk I, Mairesse J, Viltart O, Delacre M, Khalife J et al. Prenatal stress has pro-inflammatory consequences on the immune system in adult rats. Psychoneuroendocrinology 2007; 32: 114–124. 17240075 10.1016/j.psyneuen.2006.11.005

[bib75] Bronson SL, Bale TL. Prenatal stress-induced increases in placental inflammation and offspring hyperactivity are male-specific and ameliorated by maternal antiinflammatory treatment. Endocrinology 2014; 155: 2635–2646. 24797632 10.1210/en.2014-1040 PMC4060181

[bib76] Corwin EJ, Guo Y, Pajer K, Lowe N, McCarthy D, Schmiege S et al. Immune dysregulation and glucocorticoid resistance in minority and low income pregnant women. Psychoneuroendocrinology 2013; 38: 1786–1796. 23541234 10.1016/j.psyneuen.2013.02.015 PMC4082825

[bib77] Pruessner J, Wolf OT, Hellhammer DH, Buske-Kirschbaum A, von Auer K, Jobst S et al. Free cortisol levels after awakening: a reliable biological marker for the assessment of adrenocortical activity. Life Sci 1997; 61: 2539–2549. 9416776 10.1016/s0024-3205(97)01008-4

[bib78] Clow A, Thorn L, Evans P, Hucklebridge F. The awakening cortisol response: methodological issues and significance. Stress 2004; 7: 29–37. 15204030 10.1080/10253890410001667205

[bib79] World Health OrganizationMental Disorders: Glossary and Guide to their Classification in Accordance with the 9th Revision. World Health Organization Press: Geneva, Switzerland, 1978.

