# Supplementary material for: Prenatal maternal depression is associated with offspring inflammation at 25 years: a prospective longitudinal cohort study
Source: Transl Psychiatry. 2016 Nov 1;6(11):e936–. doi: 10.1038/tp.2015.155 (PMC5314108; doi:10.1038/tp.2015.155)

Supplementary Figure 1. Scatterplot of association between hs-CRP levels and maternal prenatal depression

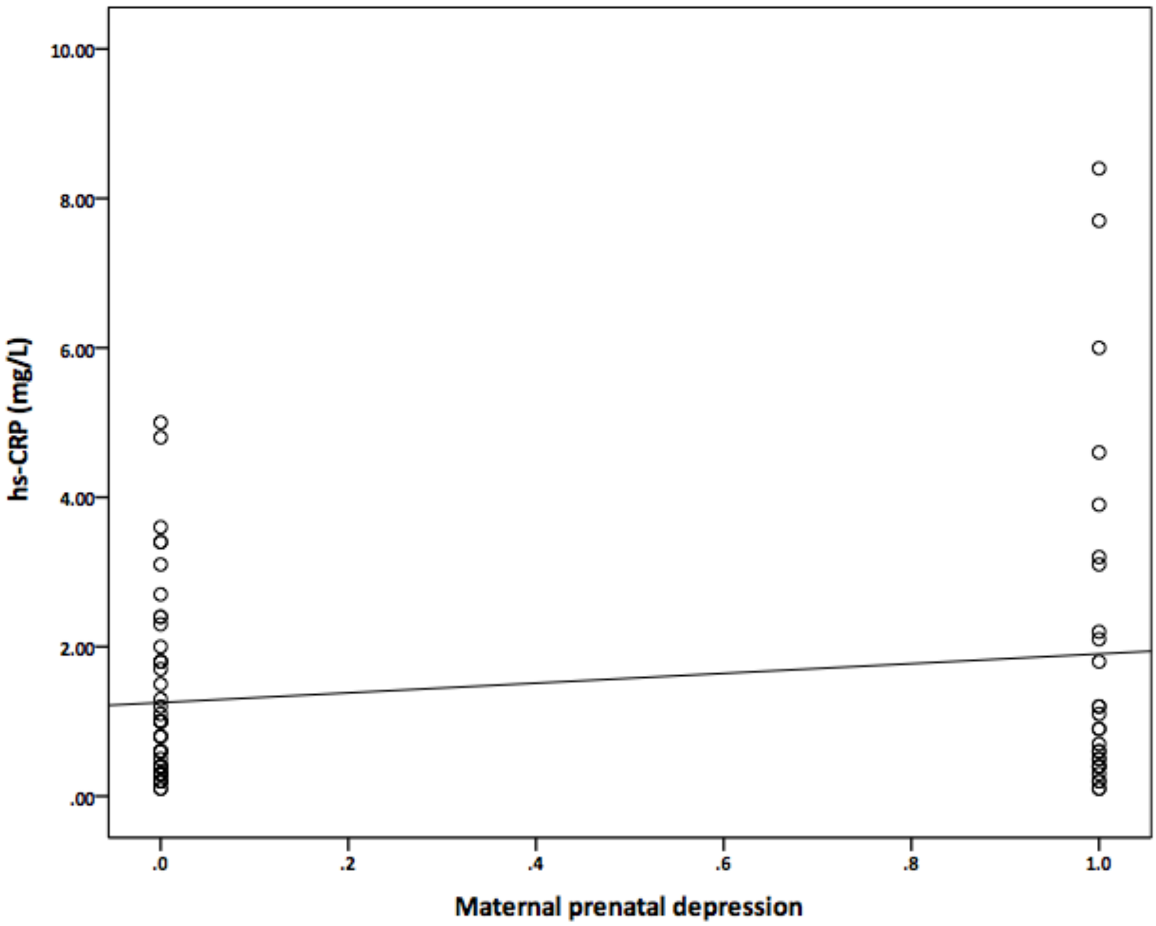

Supplement: Supplementary Figure 1 [file tp2015155x1.pdf]
