# Supplementary material for: Prenatal maternal depression is associated with offspring inflammation at 25 years: a prospective longitudinal cohort study
Source: Transl Psychiatry. 2016 Nov 1;6(11):e936–. doi: 10.1038/tp.2015.155 (PMC5314108; doi:10.1038/tp.2015.155)

Supplementary Figure 2. Scatterplot of association between cortisol awakening response and offspring child maltreatment

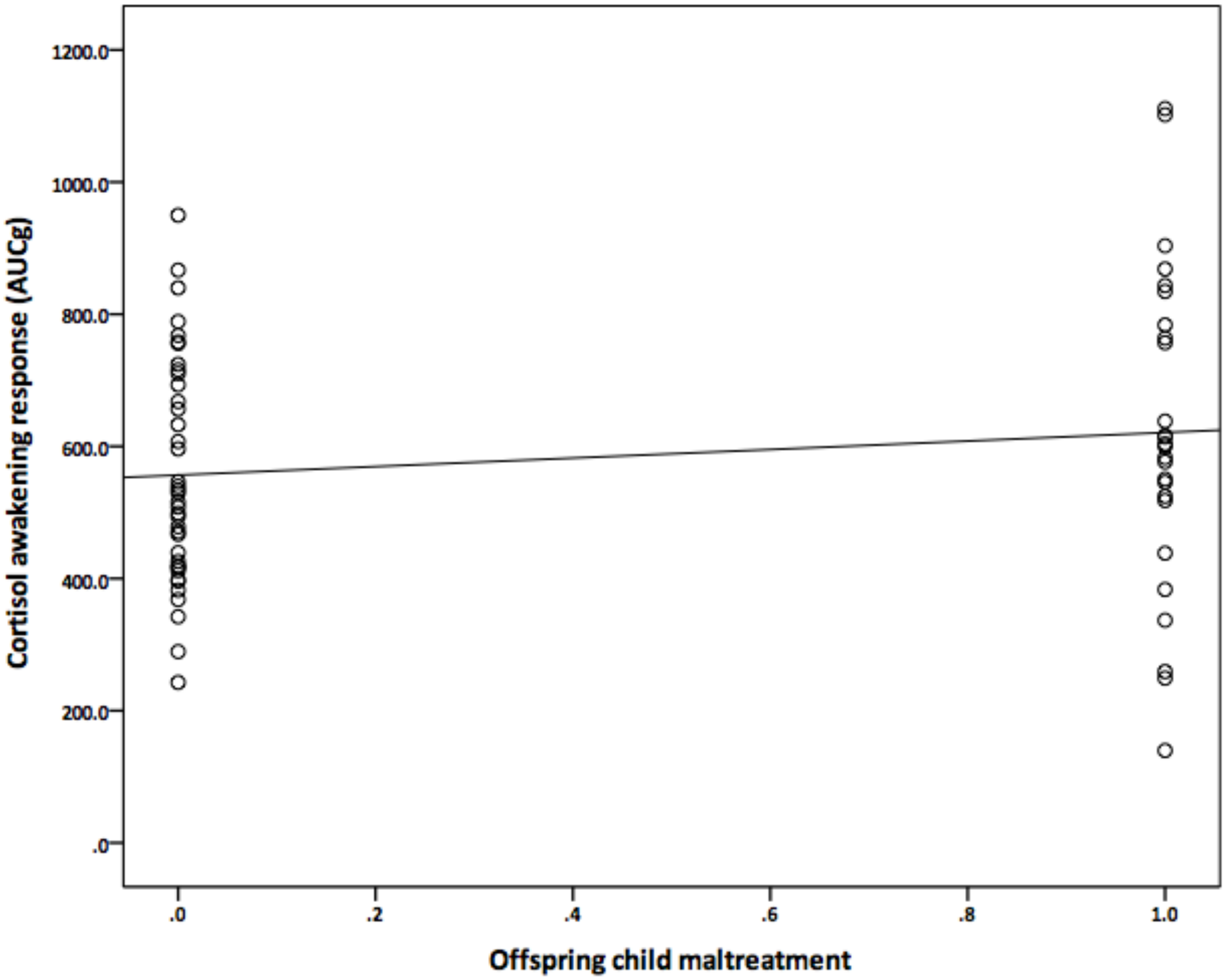

Supplement: Supplementary Figure 2 [file tp2015155x2.pdf]
